# Inclusive Leadership and Nurses’ Job Satisfaction: The Mediating Role of Team Cohesion

**DOI:** 10.1155/jonm/8135935

**Published:** 2026-10-14

**Authors:** Luay Eid Almutairi, Ahmed Mansour Almansour

**Affiliations:** ^1^ Nursing Office Almethnab General Hospital Almethnab 56526 Saudi Arabia; ^2^ Department of Nursing Administration College of Nursing Majmaah University Al Majmaah 11952 Saudi Arabia mu.edu.sa; ^3^ Department of Adult and Advanced Nursing Care College of Nursing Majmaah University Al Majmaah 11952 Saudi Arabia mu.edu.sa

**Keywords:** inclusive leadership, job satisfaction, mediation analysis, multicultural workforce, Saudi Arabia, team cohesion

## Abstract

**Background:**

Inclusive leadership is an important leadership approach that fosters supportive, collaborative, and psychologically safe work environments. Although previous research has demonstrated positive associations between inclusive leadership and nurses’ job satisfaction, limited evidence has examined the mechanisms underlying this relationship, particularly the mediating role of team cohesion in multicultural nursing workforces.

**Aim:**

To examine the mediating role of team cohesion in the relationship between inclusive leadership and nurses’ job satisfaction among registered nurses working in multicultural hospitals in Saudi Arabia.

**Methods:**

A cross‐sectional correlational study was conducted among 1015 registered nurses recruited through convenience sampling from nine hospitals in the Qassim Health Cluster, Saudi Arabia. Data were collected using the Inclusive Leadership Questionnaire, the Erlangen Team Cohesion at Work Scale, and the Job Satisfaction Scale for Nurses. Descriptive statistics, Pearson’s correlation, and Hayes’ PROCESS macro (Model 4) with 5000 bootstrap samples were used for data analysis.

**Results:**

Nurses reported high levels of inclusive leadership (*M* = 3.70 ± 0.72), moderate levels of team cohesion (*M* = 2.63 ± 0.45), and moderate levels of job satisfaction (*M* = 4.06 ± 0.54). Inclusive leadership was positively associated with team cohesion (*r* = 0.618, *p* < 0.001) and job satisfaction (*r* = 0.238, *p* < 0.001), while team cohesion was positively associated with job satisfaction (*r* = 0.390, *p* < 0.001). Mediation analysis demonstrated a significant indirect effect of inclusive leadership on nurses’ job satisfaction through team cohesion (Effect = 0.182, BootSE = 0.042, 95% bootstrap CI [0.096, 0.262]). The mediation model explained 15.2% of the variance in job satisfaction, compared with 5.6% in the total‐effect model.

**Conclusion:**

Team cohesion showed a significant indirect statistical association in the relationship between inclusive leadership and nurses’ job satisfaction in multicultural healthcare settings.

**Implications for Nursing Management:**

Strengthening nurse managers’ inclusive leadership competencies and implementing strategies that enhance team cohesion may improve nurses’ job satisfaction, collaboration, and the quality of patient care in multicultural healthcare organizations.

## 1. Introduction

Inclusive leadership is a modern, people‐oriented leadership style that is transparent, accessible, fair, respects diversity, and allows employees to participate actively in the decision‐making process [1, 2]. Inclusive leaders value varied viewpoints, and they build trust, belongingness, and psychological safety, enabling workers to participate regardless of their professional and cultural background, thereby supporting favorable organizational climates and employee well‐being [3–6]. Inclusive leadership has been investigated in a range of organizational settings but is especially relevant in healthcare, where high‐quality patient care depends on effective collaboration, communication, and coordinated decision‐making of multidisciplinary and multicultural teams [6, 7]. Recently, studies have shown that inclusive leadership can improve psychological safety, job engagement, creative work behavior, and supportive workplace climates for nurses, as well as enhance collaboration, recognition, and interpersonal relationships and, consequently, employee and organizational outcomes [4, 6, 8–11].

The organizational setting in which leadership is conducted may also impact the efficacy of leadership behaviors. In healthcare environments, when the workforce is culturally varied, disparities in language, cultural values, communication styles, and professional experience may provide significant obstacles in cooperation, collaboration, and interpersonal connections [12–14]. In such a context, inclusive leadership is of special importance, as it fosters justice, respect for variety, participation, and psychological safety among culturally mixed teams. These leadership characteristics are intended to enhance interpersonal trust and team cohesion, which will improve nurses’ job experiences and organizational results [15–18]. Therefore, the multicultural nursing workforce in Saudi Arabia and, more specifically, the hospitals in the Qassim Health Cluster are essential contextual boundary conditions for the connections between inclusive leadership, team cohesion, and nurses’ job satisfaction. Considering the extensive dependence of the Saudi healthcare system on an international and varied nursing workforce, knowledge of these links may give context‐specific evidence for nursing leadership and workforce management in multicultural healthcare organizations [13].

Job satisfaction is a multidimensional concept that expresses the nurses’ comprehensive assessment of their work experiences and work environment, which includes meaningful patient care, professional autonomy, organizational support, leadership, communication, career opportunities, and relations with colleagues [19–21]. Job satisfaction is connected with workforce stability, employee well‐being, retention, quality of care, and patient safety [22]. Hence, it is a responsibility to uncover organizational and interpersonal factors of job satisfaction. Of them, an increased interest has been attracted to team cohesion. A close‐knit nursing team is characterized by trust, common dedication, collegial solidarity, and collective accountability [23]. Such teams facilitate effective communication, joint decision‐making, coordinated patient care, and increased collaboration, leading to greater job satisfaction for nurses and improved patient outcomes.

Several studies have examined the mediating mechanisms through which inclusive leadership influences nurses’ outcomes, identifying organizational‐ and individual‐level factors. These factors included high‐performance work practices, organization‐based self‐esteem, psychological empowerment, psychological safety, and felt workgroup inclusion, which were identified as important mediators of job satisfaction, turnover intention, quality of care, and other nurses’ outcomes [8–10, 24–27]. These findings suggest that the effects of inclusive leadership are often realized indirectly through mediating mechanisms; however, these mechanisms are conceptually distinct from team cohesion. High‐performance work practices are organizational‐level mechanisms, whereas psychological constructs reflect individual employees’ perceptions and experiences. Team cohesion is instead a relational construct at the team level, and it is defined by mutual trust, collegial solidarity, shared commitment, and collaborative problem‐solving among teammates [28–30]. The collaborative character of nursing practice makes the examination of team cohesion a unique way of looking at how inclusive leadership might improve nurses’ job satisfaction by improving interpersonal connections and collaboration.

There is a growing amount of research on inclusive leadership in the healthcare context; however, critical knowledge gaps exist. First, although earlier research has shown a direct beneficial association between inclusive leadership and nurses’ job satisfaction, little research has studied how this relationship works. Second, whereas the majority of prior research on mediation has been on organizational practices or individual psychological characteristics, very little empirical work has been done on team‐level relational processes (e.g., team cohesion). Third, data from multicultural healthcare settings are scarce. The ethnically diverse nursing workforces of Saudi Arabia provide unique challenges and opportunities for leadership, cooperation, and collaboration. Therefore, it is of theoretical and practical importance to investigate the mediation effect of team cohesion on the relationship between inclusive leadership and job satisfaction of nurses in multicultural healthcare organizations.

Therefore, this study aimed to examine the relationship between inclusive leadership and nurses’ job satisfaction and to determine whether team cohesion mediates this relationship among nurses working in multicultural healthcare settings in Saudi Arabia. Based on previous empirical and theoretical evidence, we hypothesized that (H1) inclusive leadership would be positively associated with nurses’ job satisfaction; (H2) inclusive leadership would be positively associated with team cohesion; (H3) team cohesion would be positively associated with nurses’ job satisfaction; and (H4) team cohesion would mediate the relationship between inclusive leadership and nurses’ job satisfaction. Filling this gap is crucial since the mediating function of team cohesion may give a more complete account of the mechanisms via which inclusive leadership improves nurses’ job satisfaction. In addition, the study may help to enhance the current literature on nurse leadership and contribute to more complete knowledge of how inclusive leadership leads to good workplace outcomes in collaborative healthcare contexts.

## 2. Materials and Methods

### 2.1. Study Design

The current study adopted a cross‐sectional correlational design to examine the relationships among inclusive leadership, team cohesion, and nurses’ job satisfaction. The study also estimated the indirect statistical association between inclusive leadership and nurses’ job satisfaction through team cohesion. This design permits examination of associations among variables measured at a single point in time; however, because the predictor, mediator, and outcome were assessed concurrently, it cannot establish temporal precedence or causal mediation. Therefore, the mediation analysis in this study was interpreted as an assessment of an indirect statistical association rather than evidence of a causal pathway [31].

### 2.2. Setting

This study was conducted in nine hospitals affiliated with the Qassim Health Cluster, Saudi Arabia. These are tertiary and general hospitals that employ nurses from diverse cultural and professional backgrounds, reflecting the multicultural nature of the Saudi healthcare workforce. This setting provides an appropriate context for examining the relationships among inclusive leadership, team cohesion, and nurses’ job satisfaction within multicultural nursing teams.

### 2.3. Sample Size Calculation

The estimated necessary sample size for multiple linear regression was calculated using the G∗Power program. The regression equation predicting job satisfaction included inclusive leadership and team cohesion as two factors. The sample size was computed for a medium effect size (*f* ^2^ = 0.15), a significance level (*α*) of 0.05, and a statistical power of 0.95. The final analytic sample comprised 1015 registered nurses, which was much more than the minimum required sample size and provided acceptable accuracy for the intended analyses.

### 2.4. Sampling and Participants

A convenience sampling technique was used to recruit registered nurses from the nine participating hospitals. Nursing directors at each hospital facilitated dissemination of the electronic questionnaire through institutional communication channels to nurses who potentially met the eligibility criteria. Recruitment remained open simultaneously across the participating hospitals throughout the data‐collection period, and eligible nurses who received or accessed the questionnaire could voluntarily participate. Accordingly, the sample comprised eligible nurses who were accessible and chose to respond during the recruitment period; therefore, the recruitment strategy did not constitute a census of the eligible nursing population. Because convenience sampling was used, proportional allocation according to hospital size was not performed.

The inclusion criteria were as follows:

• Registered nurses employed in one of the participating hospitals.
• Currently providing direct patient care.

The exclusion criteria were as follows:

• Nursing students or interns.
• Administrative nurses not directly involved in patient care.

### 2.5. Study Measurement Tools

#### 2.5.1. Participants’ Demographics Questionnaire

The participants completed a demographics questionnaire including age, gender, marital status, educational level, working department, and years of professional experience. Marital status was included because some previous studies have reported its potential association.

#### 2.5.2. Inclusive Leadership Questionnaire (ILQ)

The second measurement tool of the study was the ILQ, which helps to determine the attitude toward inclusive leadership behaviors in organizations [32]. This tool is used to assess the leader in terms of them addressing equality, belonging, and acknowledgment of individual distinctiveness. The tool has 40 items that are grouped into four dimensions: (i) providing equal opportunities and fair treatment to all work unit members; (ii) encouraging integration of and synergy among all work unit members; (iii) directly addressing work unit members’ fundamental needs for uniqueness, authenticity, and belongingness; and (iv) implementing organizational diversity and inclusion‐related policies and programs in the work unit. Responses are rated using a 5‐point Likert scale ranging from 1 (*almost never*) to 5 (*almost always*), with higher scores indicating greater perceived inclusive leadership. Mean scores were interpreted as low (1.00–2.33), moderate (2.34–3.67), and high (3.68–5.00). This tool has good internal consistency, with a reported reliability coefficient of 0.97, and it was adopted into this study without modification [32]. In this study, the Cronbach’s alpha for ILQ using the final analytic sample was 0.98, which demonstrated an excellent internal consistency.

#### 2.5.3. Erlangen Team Cohesion at Work Scale (ETC)

The third measurement tool of the study was the ETC, which is an instrument for assessing cohesion in professional teams [33]. This tool has 13 items that are grouped into two dimensions: (i) collegial solidarity and (ii) unity and problem management. Responses are rated using a 5‐point Likert scale ranging from 0 (*totally disagree*) to 4 (*totally agree*), with higher scores indicating stronger team cohesion. Mean scores were interpreted as low (0.00–1.33), moderate (1.34–2.67), and high (2.68–4.00). The tool is very reliable, with a reported internal consistency coefficient of 0.91, and it was adopted into this study without modification [33]. In this study, the Cronbach’s alpha for ETC using the final analytic sample was 0.96, which demonstrated an excellent internal consistency.

#### 2.5.4. Job Satisfaction Scale for Nurses (JSS)

The last measurement tool of the study was the JSS, which was created to measure the level of satisfaction of employees in relation to various work‐related variables [34]. There are 24 items in this tool, which are grouped under seven factors: administration, co‐workers, career, patient care, relationship with supervisor, nursing education, and communication. Items are scored on a 7‐point Likert scale ranging from 1 (*strongly disagree*) to 7 (*strongly agree*), with higher scores indicating greater job satisfaction. Mean scores were interpreted as low (1.00–3.00), moderate (3.01–5.00), and high (5.01–7.00). The original instrument demonstrated acceptable reliability, with a reported internal consistency coefficient of 0.85, and it was adopted into this study without modification [34]. In this study, the Cronbach’s alpha for JSS using the final analytic sample was 0.97, which demonstrated an excellent internal consistency.

### 2.6. Validity, Reliability, and Pilot Study

The instruments used in this study are internationally recognized instruments with strong psychometric properties in prior validation studies [35–37]. To ensure contextual appropriateness, all tools were reviewed by a panel of three experts in nursing leadership and management, confirming their content appropriateness and relevance to the study setting.

Before the main investigation, a pilot study was performed with 80 registered nurses from different hospitals, who were not included in the final study sample analysis. In the pilot research, the questionnaire was assessed for clarity, readability, appropriateness, and time to complete. Internal consistency reliability of the pilot study was evaluated using Cronbach’s alpha coefficients. These Cronbach’s alpha coefficients were 0.89 for inclusive leadership, 0.83 for team cohesion, and 0.84 for job satisfaction, which all indicate satisfactory reliability for all study instruments.

### 2.7. Data Collection

Nursing directors at the participating hospitals facilitated dissemination of the electronic questionnaire through institutional communication channels. An anonymous online link to the study questionnaire containing an electronic consent form and information about the study was sent to the directors to send on to the nurses. The questionnaire was administered in the English language. All information about the study and contact details for the researcher were provided at the start of the electronic form. There was also a clear statement of the right of the subjects to withdraw from the study at any time. The questionnaire completion time was between 15 and 20 min, and the data were collected between February 2026 and April 2026. Although nursing directors facilitated questionnaire dissemination, this recruitment approach may have created a perceived sense of obligation among some nurses and is therefore acknowledged as a potential source of participation and social‐desirability bias.

### 2.8. Ethical Considerations

This study was performed in line with the ethical principles outlined in the Declaration of Helsinki and institutional research guidelines. Prior to conducting the study, ethical approval was obtained from the Regional Research Ethics Committee in Qassim Province, Saudi Arabia (approval number: 607‐47‐011549). Electronic and verbal permissions were also granted by the individual hospitals. No personal information was recorded, and the confidentiality and anonymity of the participants were ensured. Participation was voluntary, and the respondents were free to withdraw from the study at any point without penalty. The information was stored safely and was only available to the researchers, in line with the ethical principles of beneficence, non‐maleficence, and respect for individuals.

### 2.9. Data Analysis

Prior to data analysis, the dataset was screened for completeness. Questionnaires with incomplete responses were excluded from the analysis. The data were analyzed using IBM SPSS Statistics (version 27.0). Descriptive statistics, including frequencies, percentages, means, and standard deviations, were employed to summarize the participants’ demographic characteristics and their scores for the study variables. Cronbach’s alpha coefficients were calculated to assess the internal consistency and reliability of the scales. Mean scores for each study variable were interpreted according to the predefined classification ranges described under the corresponding measurement instrument. Pearson’s correlation coefficients were utilized to analyze the relationships between the study variables. To test the mediation model, the PROCESS macro (Version 4.2) for SPSS was used (Model 4). This procedure was employed to explore the mediating role of team cohesion in the relationship between inclusive leadership and nurses’ job satisfaction. Unstandardized regression coefficients (B) are reported for the PROCESS regression models. The significance of the indirect effect was assessed using a bias‐corrected bootstrapping method with 5000 resamples and 95% confidence intervals (CIs); the indirect effect is reported as Effect with its bootstrap standard error (BootSE) and bootstrap CI. An effect was considered statistically significant if the 95% CI did not include zero. Overall statistical significance for all tests was determined at a two‐tailed *p* value < 0.05.

## 3. Results

### 3.1. Participants’ Characteristics and Comparison of Study Variables

A total of 1015 nurses participated in this study. The majority were over 30 years of age (60.3%), female (59.3%), and married (58.0%). Regarding educational level, the most prevalent qualification was a bachelor’s degree (71.9%). In terms of working department, the largest group worked in critical care (18.8%). Regarding professional tenure, the sample was primarily composed of nurses with 1–5 years (37.3%) and 6–10 years (37.3%) of experience.

Comparative analysis revealed that there were no statistically significant differences in nurses’ perceptions of inclusive leadership, team cohesion, or job satisfaction according to age, gender, or marital status (all *p* > 0.05). However, significant differences were observed in other demographic variables (Table 1). Team cohesion differed significantly by educational level (*F* = 2.81, *p* = 0.038), with bachelor’s degree holders reporting higher scores than those with a PhD; however, this result should be interpreted with caution due to the small PhD subgroup size (*n* = 18), which may limit the stability and generalizability of this subgroup estimate. Job satisfaction also varied significantly across working departments (*F* = 8.09, *p* < 0.001), with nurses in the “Other” category reporting substantially lower levels (*M* = 2.98) than those in other units; this finding should be interpreted with caution given the very small sample size of this subgroup (*n* = 11), which may render the estimate unstable. Additionally, years of professional experience significantly influenced both inclusive leadership (*F* = 2.65, *p* = 0.032) and team cohesion (*F* = 2.70, *p* = 0.030). Post hoc analyses showed that nurses with less than 1 year of experience scored significantly lower on both variables compared to those with more experience (Table 1).

**TABLE 1:** Sample characteristics and comparison of the study variables (*N* = 1015).

Characteristic	*N* (%)	Inclusive leadership		Team cohesion		Job satisfaction	
		Mean (SD)	*F* (*p*)	Mean (SD)	*F* (*p*)	Mean (SD)	*F* (*p*)
*Education level*							
Diploma	137 (13.5)	3.66 (0.77)	0.96 (0.413)	2.58 (0.48)	2.81 (0.038)	4.01 (0.41)	2.17 (0.090)
Bachelor’s	730 (71.9)	3.73 (0.70)		2.66 (0.43)		4.09 (0.50)	
Master’s	130 (12.8)	3.67 (0.70)		2.60 (0.49)		3.98 (0.71)	
PhD	18 (1.8)	3.51 (0.93)		2.44 (0.72)		3.97 (1.06)	

*Working department*							
Medical–surgical	165 (16.3)	3.69 (0.74)	0.31 (0.931)	2.58 (0.43)	1.14 (0.336)	4.05 (0.48)	8.09 (< 0.001)
Critical care	191 (18.8)	3.71 (0.73)		2.65 (0.44)		4.07 (0.61)	
Emergency	181 (17.8)	3.67 (0.66)		2.64 (0.46)		4.07 (0.60)	
Pediatrics	150 (14.8)	3.77 (0.67)		2.68 (0.42)		4.12 (0.41)	
Obstetrics & gynecology	149 (14.7)	3.70 (0.74)		2.60 (0.43)		4.04 (0.32)	
Outpatient/ambulatory	168 (16.6)	3.72 (0.73)		2.67 (0.48)		4.09 (0.50)	
Other	11 (1.1)	3.71 (1.06)		2.56 (1.10)		2.98 (1.53)	

*Years of professional experience*							
< 1	54 (5.3%)	3.42 (0.81)	2.65 (0.032)	2.46 (0.68)	2.70 (0.030)	3.86 (1.01)	2.39 (0.050)
1–5	379 (37.3)	3.72 (0.70)		2.64 (0.44)		4.08 (0.51)	
6–10	379 (37.3)	3.74 (0.72)		2.67 (0.43)		4.08 (0.43)	
11–15	132 (13.0)	3.67 (0.76)		2.61 (0.43)		4.07 (0.45)	
> 15	71 (7.0)	3.74 (0.63)		2.65 (0.48)		4.00 (0.79)	

### 3.2. Descriptive Statistics of the Study Variables

Table 2 presents the means and standard deviations for the study variables and their respective dimensions. As indicated in the table, the total mean score for inclusive leadership was 3.70 ± 0.72 (out of 5), which falls within the high category according to the predefined scoring criteria, suggesting a high level of inclusive leadership practices as perceived by the staff nurses. Among its dimensions, “Encouraging integration and synergy” (*M* = 3.71, SD = 0.72) and “Implementing organizational diversity policies” (*M* = 3.71, SD = 0.78) received the highest scores. Conversely, “Providing equal opportunity” (*M* = 3.70, SD = 0.73) and “Addressing uniqueness and belonging” (*M* = 3.70, SD = 0.74) scored slightly lower.

**TABLE 2:** Descriptive statistics for the study variables and their dimensions (N = 1015).

Variable/dimension	*M*	SD
Inclusive leadership	3.70	0.72
Providing equal opportunity and fair treatment to all work unit members	3.70	0.73
Encouraging integration of and synergy among all work unit members	3.71	0.72
Directly addressing work unit members’ fundamental needs for uniqueness, authenticity, and belonging	3.70	0.74
Implementing organizational diversity‐ and inclusion‐related policies and programs in the work unit	3.71	0.78
Team cohesion	2.63	0.45
Collegial solidarity	2.90	0.60
Unity and problem management	2.40	0.40
Job satisfaction	4.06	0.54
Administration	4.41	0.76
Co‐workers	3.98	0.61
Career	4.31	0.70
Patient care	3.13	1.04
Relationship with supervisor	3.98	0.62
Nursing education	3.70	0.69
Communication	4.86	1.08

*Note: M*, mean.

Abbreviation: SD, standard deviation.

Additionally, the results showed that the total mean score for perceived team cohesion was 2.63 ± 0.45 (out of 4), which falls within the moderate category according to the predefined scoring criteria, indicating a moderate level of perceived unity within the work units. When examining its subdimensions, “Collegial solidarity” scored higher (2.90 ± 0.60), while “Unity and problem management” scored lower (2.40 ± 0.40). Finally, the mean score for overall job satisfaction was 4.06 ± 0.54 (out of 7), which falls within the moderate category according to the predefined scoring criteria, reflecting a moderate level of satisfaction among the participants. Among its seven dimensions, “Communication” received the highest satisfaction score (4.86 ± 1.08), while “Patient care” scored the lowest (3.13 ± 1.04), indicating relatively lower satisfaction in this domain compared to other aspects of nursing work.

### 3.3. Correlation of Study Variables

Table 3 presents the Pearson correlation coefficients among the study variables. This table shows that inclusive leadership had significant positive relationships with team cohesion (*r* = 0.618, *p* < 0.001) and job satisfaction (*r* = 0.238, *p* < 0.001). In addition, team cohesion and job satisfaction were significantly positively related (*r* = 0.390, *p* < 0.001).

**TABLE 3:** Pearson correlation matrix of the study variables (*N* = 1015).

Variable	Inclusive leadership	Team cohesion	Job satisfaction
Inclusive leadership	1	0.618	0.238
Team cohesion	0.618	1	0.390
Job satisfaction	0.238	0.390	1

*Note:* Values below the diagonal are Pearson correlation coefficients. All correlations were statistically significant at *p* < 0.001.

### 3.4. Mediation Analysis

We applied the Hayes PROCESS macro (Model 4) to evaluate the study hypotheses. First, the total association between inclusive leadership and nurses’ job satisfaction was examined. Inclusive leadership was significantly associated with job satisfaction (*B* = 0.178, SE = 0.023, *t* = 7.78, *p* < 0.001, 95% CI [0.133, 0.223]), and the total‐effect model accounted for 5.6% of the variance in job satisfaction (*R* ^2^ = 0.056). Subsequently, the indirect statistical association through team cohesion was assessed using 5000 bootstrap samples and 95% CIs. Inclusive leadership was positively associated with team cohesion (*B* = 0.392, SE = 0.016, *t* = 24.99, *p* < 0.001, 95% CI [0.362, 0.423]). In the outcome model, team cohesion was positively associated with nurses’ job satisfaction (*B* = 0.464, SE = 0.043, *t* = 10.66, *p* < 0.001, 95% CI [0.378, 0.549]).

The bootstrapped indirect effect of inclusive leadership on job satisfaction through team cohesion was statistically significant (Effect = 0.182, BootSE = 0.042, 95% bootstrap CI [0.096, 0.262]), because the confidence interval did not include zero. After team cohesion was included in the outcome model, the direct association between inclusive leadership and job satisfaction was not statistically significant (*B* = −0.004, SE = 0.028, *t* = −0.13, *p* = 0.896, 95% CI [−0.058, 0.051]). The significant bootstrapped indirect effect, together with the nonsignificant direct effect, was consistent with an indirect‐only statistical mediation pattern. Given the cross‐sectional design, this finding should not be interpreted as evidence of a temporally ordered or causal mediation process. Taken together, inclusive leadership and team cohesion explained 15.2% of the variance in nurses’ job satisfaction (*R* ^2^ = 0.152; Table 4).

**TABLE 4:** Direct, indirect, and total effects analysis of the mediation model variables (*N* = 1015).

Effect/path	*B*/effect	SE/BootSE	*t*	*p*	95% CI
					Lower/upper
Inclusive leadership to team cohesion	0.392	0.016	24.99	< 0.001	0.362/0.423
Team cohesion to job satisfaction	0.464	0.043	10.66	< 0.001	0.378/0.549
Inclusive leadership to job satisfaction	−0.004	0.028	−0.13	0.896	−0.058/0.051
Indirect effect of inclusive leadership on job satisfaction	0.182	0.042	—	—	0.096/0.262
Total effect	0.178	0.023	7.78	< 0.001	0.133/0.223

*Note:* B = unstandardized regression coefficient; BootSE = bootstrap standard error. The indirect effect was estimated using 5000 bias‐corrected bootstrap resamples. Model *R* ^2^ values were 0.382 for the mediator model, 0.056 for the total‐effect model, and 0.152 for the outcome model including inclusive leadership and team cohesion. Because the variables were measured concurrently, the indirect effect represents a statistical indirect association rather than evidence of temporal or causal mediation.

Abbreviations: CI, confidence interval; SE, standard error.

## 4. Discussion

The aim of this research was to investigate the links between inclusive leadership, team cohesion, and job satisfaction of nurses and to assess the mediating function of team cohesion among registered nurses in nine hospitals in the Qassim Health Cluster, Saudi Arabia. The nurses indicated high levels of inclusive leadership, moderate levels of team cohesion, and moderate levels of job satisfaction. Inclusive leadership was favorably related to team cohesion and job satisfaction. Team cohesion was positively associated with job satisfaction, and the statistical analysis demonstrated a significant indirect association between inclusive leadership and nurses’ job satisfaction through team cohesion. Given the cross‐sectional design, this indirect association should not be interpreted as evidence of temporal or causal mediation. These results imply open, accessible, fair, and inclusive leadership behaviors may be associated with more favorable nurses’ job experiences and stronger interpersonal interactions within nursing teams [25, 26, 37–40].

The highest level of inclusive leadership suggests that nurse managers typically displayed openness, accessibility, fairness, and staff engagement, but these behaviors might be further enhanced. This corresponds with studies showing that inclusive leadership promotes participation, respect, and supportive relationships, which in turn improves nurses’ psychological safety, innovative work behavior, job satisfaction, and workplace engagement [8–11, 41]. The highest score within the Saudi healthcare system may be attributed to the ongoing development of collaborative leadership practices with organizational change and changing expectations of nurse managers [42–44]. Therefore, leadership development is essential to increase inclusive communication, cultural responsiveness, staff engagement, and psychological safety.

Nurses also indicated moderate team cohesion, indicating generally good working relationships but opportunities for growth such as mutual trust, shared commitment, collaborative problem‐solving, and collective responsibility in clinical teams. Good communication, coordinated decision‐making, continuity of patient care, and workplace health are connected to cohesive teams. Consistent with the results, Meneses‐La‐Riva et al. [45] showed that increased team cohesion was associated with improved communication, cooperation, and team functioning. Supportive interpersonal ties have also been associated with positive psychological outcomes and lower turnover intention among nurses [46, 47]. Importantly, the current research conceptualizes team cohesion as a relational process at the team level, rather than an organizational practice or individual psychological condition. Previous mediation studies have focused on high‐performance work practices, organization‐based self‐esteem, psychological safety, or self‐efficacy. Team cohesion is the strength of connections and cooperation among workgroup members. This is especially true in nursing, where safe and effective treatment relies significantly on communication, cooperation, and shared responsibility.

Overall job satisfaction was moderate, which suggests that nurses were generally content with their work but were still facing organizational and clinical issues. This conclusion is consistent with data indicating that organizational support, leadership, collaboration, workload, and communication are critical factors that influence nurses’ experiences at work [48, 49]. The job satisfaction facet of communication was rated the highest, suggesting rather positive opinions of communication in the workplace. Effective communication promotes teamwork, eliminates misconceptions, and enhances patient safety and staff satisfaction [45]. The Patient Care dimension had the lowest score (3.13 ± 1.04), showing somewhat less satisfaction with areas of work directly associated with care delivery. The present study did not measure the specific organizational or clinical factors responsible for this comparatively lower score; therefore, its underlying causes cannot be determined from the current data. Previous research has identified factors such as increasing patient acuity, staffing shortages, high workloads, time pressure, limited resources, and adverse work environments as potential influences on nurses’ experiences of patient care [22]. These factors should therefore be considered possible contextual explanations rather than explanations established by the present study. Future research should directly measure staffing, workload, patient acuity, resources, and other work‐environment characteristics to determine whether they contribute to nurses’ satisfaction with patient care.

The correlation investigations further validated the expected relationships. Inclusive leadership showed the strongest bivariate association with team cohesion, suggesting that inclusive leaders may build an environment of trust, mutual respect, psychological safety, and participative decision‐making that may lead to collaboration and shared goals. This association may be particularly essential in multicultural healthcare settings, as nurses operate across cultural, language, and professional barriers. Likewise, positive and inclusive leadership has been linked to better collegial connections, cooperation, collaborative practice, and patient safety culture [6]. Team cohesion was also positively associated with nurses’ job satisfaction, emphasizing the importance of support, trust, and collaboration in shaping nurses’ professional experiences. The nurses who reported more collaboration in their teams also reported reduced occupational stress and more organizational commitment and job satisfaction. These findings demonstrate meaningful bivariate relationships among inclusive leadership, team cohesion, and job satisfaction; however, their direction and temporal sequence cannot be established from the cross‐sectional data.

The main contribution of the present analysis is the identification of a significant indirect statistical association between inclusive leadership and nurses’ job satisfaction through team cohesion. Nurses who perceived greater inclusive leadership also reported greater team cohesion, and greater team cohesion was associated with higher job satisfaction. The bootstrapped confidence interval for the indirect effect did not include zero, indicating that the indirect association was statistically significant. Following Hayes and Preacher [50], interpretation of the statistical mediation analysis was based primarily on the bootstrapped indirect effect rather than on the non‐significance of the direct effect. However, because inclusive leadership, team cohesion, and job satisfaction were measured concurrently, the findings do not establish the temporal ordering required for causal mediation. Therefore, the findings are consistent with the possibility that nurses who perceive more inclusive leadership also perceive greater team cohesion and that these perceptions are associated with higher job satisfaction. Longitudinal or prospective research with temporally separated measurements is needed to determine whether team cohesion temporally mediates the relationship between inclusive leadership and job satisfaction. The total‐effect model explained 5.6% of the variance in job satisfaction, whereas the model including team cohesion explained 15.2%, indicating that the inclusion of team cohesion increased the statistical explanatory capacity of the model without establishing a causal pathway.

The findings might be explained theoretically in terms of Social Exchange Theory. Blau [51] noted that helpful exchanges based on fairness, accessibility, respect, and assistance may be related to higher trust, perceived organizational support, cooperation, and better interpersonal connections. From this theoretical view, the observed connections of inclusive leadership, team cohesion, and job satisfaction may be indicative of relational exchange processes although not temporally explored in the current research. Another important theoretical approach is the social identity theory. Inclusive leaders appreciate individual contributions, but they also promote a feeling of community and inclusion. From this point of view, higher identification with the nursing team may be related to increased trust, commitment, collective responsibility, and job satisfaction [52]. These theoretical processes may be especially significant for multicultural teams, since inclusive leadership may be helpful in bridging cultural gaps and creating shared professional identity.

Nurses with less than 1 year of professional experience showed poorer opinions of inclusive leadership and team cohesion than their counterparts. Transition shock, which is common among new graduate/new hire nurses who suffer uncertainty, stress, and difficulties transitioning to professional obligations and organizational expectations, might be part of the explanation for this result [53, 54]. Transition shock, however, should not be the sole reason. Differences in educational preparation, professional aspirations, and past experience may also affect views between cohorts. Novice nurses may still be in the throes of organizational socialization and integrating into existing teams. The ways in which novice and experienced nurses report may vary, as may expectations of leadership. Because the research was cross‐sectional, it is not possible to discern definitively between these theories. More longitudinal work is required to better understand the evolution of views of leadership and cohesiveness in the early professional development of nurses.

Finally, the results should be viewed in the particular multicultural setting of healthcare in Saudi Arabia. Saudi hospitals employ nurses from different national, linguistic, educational, and cultural backgrounds, which provides not only opportunities for information exchange but also possible obstacles in communication, cultural understanding, collaboration, and interpersonal interactions. Evidence of these multicultural dynamics was reported in Theron et al. [13], therefore highlighting the importance of leadership approaches in supporting inclusion, psychological safety, communication, and collaboration in Saudi healthcare hospitals.

## 5. Strengths and Limitations

To the best of our knowledge, this study is among the first to evaluate team cohesion as a team‐level relational mediator between inclusive leadership and nurses’ job satisfaction in a multicultural healthcare context. The large sample of 1015 registered nurses from nine hospitals, the use of validated instruments, and mediation analysis using Hayes’ PROCESS macro strengthened the study. However, the cross‐sectional design does not establish temporal ordering among inclusive leadership, team cohesion, and job satisfaction and therefore does not permit causal conclusions regarding the mediation pathway. In addition, the use of self‐administered surveys may have introduced response and social‐desirability bias. Despite our emphasis on anonymity, confidentiality, voluntary participation, and withdrawal without penalty, recruitment was facilitated by nursing directors, which might have resulted in a perceived obligation or biased some nurses to give more positive responses, especially regarding leadership‐related metrics. Thus, the prospect of perceived compulsion and socially desirable behavior cannot be fully ruled out. Furthermore, convenience sampling and the small sizes of certain subgroups, particularly PhD nurses and nurses in the “Other” department, may limit the representativeness and generalizability of the findings; therefore, these results should be interpreted with caution.

## 6. Conclusion

This study demonstrated that inclusive leadership was positively associated with team cohesion and nurses’ job satisfaction among registered nurses working in multicultural hospitals in Saudi Arabia. The analysis identified a significant indirect statistical association between inclusive leadership and job satisfaction through team cohesion, highlighting team cohesion as a potentially important relational mechanism linking leadership behaviors with nurses’ workplace outcomes. Given the cross‐sectional design, this finding does not establish temporal or causal mediation. These findings contribute to the growing evidence on inclusive leadership in multicultural healthcare settings.

## 7. Implications for Nursing Management

The results have some key implications for nursing management. Given the significant positive associations between inclusive leadership and team cohesion (*B* = 0.392, *p* < 0.001) and between team cohesion and nurses’ job satisfaction (*B* = 0.464, *p* < 0.001), healthcare organizations should develop nurse managers’ inclusive leadership competencies through training programs on open communication, accessibility, fairness, active listening, diversity appreciation, and participative decision‐making, while adopting team‐building, mentorship, shared governance, and interprofessional collaboration initiatives to improve team cohesion. Newly hired nurses expressed a poorer view of inclusive leadership and team cohesion, and hence extensive orientation, mentoring, and peer‐support programs should aid their integration into multidisciplinary teams. Moreover, the relatively low satisfaction with the “Patient Care” dimension reflects the need for appropriate staffing, supportive work environments, reasonable workloads, and sufficient organizational resources to provide high‐quality, patient‐centered care and enhance nurses’ job satisfaction.

## Funding

The authors extend the appreciation to the Deanship of Postgraduate Studies and Scientific Research at Majmaah University for funding this research work through the project number (R‐2026‐447).

## Conflicts of Interest

The authors declare no conflicts of interest.

## Data Availability

The data that support the findings of this study are available from the corresponding author upon reasonable request.
